# Serum IL-6 predicts immunotherapy-related adverse and outcome in advanced gastric and esophageal cancer patients with Anti-PD-1 treatment

**DOI:** 10.3389/fimmu.2025.1553882

**Published:** 2025-05-30

**Authors:** Hongfang Ma, Shasha Zhang, Pengqing Jiao, Haixia Ding, Fujun Wang, Yue Zhao, Jianhua Wu, Zhanjun Guo

**Affiliations:** ^1^ Department of Rheumatology and Immunology, The Fourth Hospital of Hebei Medical University, Shijiazhuang, China; ^2^ Department of Endocrinology, The Fourth Hospital of Hebei Medical University, Shijiazhuang, China; ^3^ Department of Gastroenterology and Hepatology, The Fourth Hospital of Hebei Medical University, Shijiazhuang, China; ^4^ Animal Center, The Fourth Hospital of Hebei Medical University, Shijiazhuang, China

**Keywords:** gastric cancer, esophageal cancer, immune checkpoint inhibitor, interleukin-6, immune-related adverse events, prognosis

## Abstract

**Purpose:**

Immune checkpoint inhibitors (ICIs) significantly prolong the survival of cancer patients. including gastric adenocarcinoma (GAC) and esophageal squamous cell carcinoma (ESCC) patients. Immune-related adverse events (irAEs) are inevitably involved in ICIs treatment sometimes with severe consequences. Extreme caution is necessary for predicting irAEs and precisely screening of appropriate patients. We evaluated the association of interleukin-6 (IL-6) with irAEs and their impacts on ICIs treatment effectiveness in advanced GAC and ESCC patients.

**Methods:**

This retrospective study analyzed 121 patients treated with ICIs between March 1, 2020 and August 31, 2023 to evaluate the association between serum IL-6 and ICIs treatment effectiveness. The occurrence of irAEs, including grade and category, and effectiveness of immunotherapy, including objective remission rate (ORR), disease control rate (DCR), progression-free survival (PFS) and overall survival (OS), was evaluated. Categorical count data were tested by chi-square test. Nonparametric rank sum tests were performed using Wilcoxon and Kruskal-Wallis test. Survival rate estimation and survival curves were generated using Kaplan–Meier curve and Log-rank test. Univariate and multivariable COX regression analyses were performed to identify independent prognostic factors.

**Results:**

A total of 121 patients including 79 with GAC and 42 with ESCC patients were randomly divided into TC (n=81) and VC (n=40) groups. Higher serum IL-6 levels were associated with increased incidence of irAEs, the outcome analysis also indicated its association with lower DCR, shorter PFS and shorter OS in TC group. The higher IL-6 related irAEs occurrence and poor prognosis (DCR, PFS) was confirmed in the VC group. Individual tumor analysis showed that higher IL-6 was associated with both irAEs occurrence and poor prognosis (DCR, PFS, OS) in ESCC patients, and with irAEs occurrence and poor prognosis (DCR, PFS) in GAC patients. No statistically significant associations were observed between pathological biomarkers including programmed cell death ligand 1 (PD-L1), mismatch repair (MMR) and human epidermal growth factor receptor 2 (HER2) and either IL-6 levels or irAEs occurrence in both GC and ESCC patients.

**Conclusion:**

Elevated serum IL-6 levels were associated with the incidence of irAEs, and higher IL-6 levels predicted worse prognosis in GAC and ESCC patients with ICIs treatment.

## 1 Introduction

Immune checkpoint inhibitors (ICIs) blocking cytotoxic T-lymphocyte antigen 4 (CTLA-4), programmed cell death 1 (PD-1) or its ligand of programmed cell death ligand 1(PD-L1) to enhance anti-tumor immunity has made a major breakthrough in cancer treatment. PD-1 on the surface of various immunocyte can bind with PD-L1 on the tumor cell to inhibit T cell activation (1, 2). ICIs combining with chemotherapy for advanced gastric cancer (GC) have moved from the third-line treatment to the first-line treatment due to their efficiency to improve overall survival (OS), progression-free survival (PFS) and objective response rate (ORR) when compared with those of chemotherapy alone (3, 4). Similarly, ICIs combining with chemotherapy for advanced esophageal cancer (EC) also transformed from the second line treatment to the first line treatment with significant improved outcomes (5–7).

Despite these breakthroughs, the overall ORR for immunotherapy in advanced EC and GC remains below 50%. Moreover, the immune system’s natural defense against cancer through ICIs regulation unavoidably results in the damage of normal tissues via abnormal stimulation of the immune system, which is called as immune-related adverse events (irAEs) (8). The occurrence of irAEs is related to the unbalance of immune homeostasis, generation of autoantibodies and autoantigens, dysbacteriosis and cytokines release (9). The types of irAEs also vary with different organs and tissues involved upon different ICIs treatment (10, 11). IrAEs seem to be associated with better effectiveness referring to ORR, PFS and OS (12–15). But serious adverse events may lead to discontinuation treatment, frequent hospitalization with immunosuppressant treatment, and even fatal (16). Therefore, identifying reliable biomarkers for precisely predicting both therapeutic efficacy and irAEs represents a critical challenge in current gastrointestinal cancer immunotherapy.

Interleukin-6 (IL-6) is involved in cell growth, survival, inflammation and immune regulation (17). It could initiate both carcinogenesis and tumor progress via various signaling pathways in tumor microenvironment (18, 19). IL-6 can increase the vascular endothelial growth factor (VEGF) expression via the Janus tyrosine kinase/signal transducer and activator of transcription 3 (JAK/STAT3) signaling pathway, thereby promoting growth, invasion and lymphangiogenesis in GC patients (20). In addition, IL-6 interacts with both epidermal growth factor receptor (EGFR) to promote immunosuppressive microenvironment by inducing myeloid-derived suppressor cells (MDSC) and the vascular endothelial growth factor receptor (VEGFR) to drive tumor-induced angiogenesis in EC (21, 22). Emerging evidence demonstrates a significant association between IL-6 levels and ICIs outcomes. In advanced lung cancer patients receiving anti-PD-1 therapy, the low baseline IL-6 levels in peripheral blood correlated with better treatment effectiveness (23, 24). IL-6 blockade could improve ICIs induced antitumor efficacy in melanoma patients (25).

IL-6 expression had been proved to be associated with irAEs occurrence in some types of cancers. IL-6 levels displayed a positive correlation with irAEs related intestinal toxicity during immunotherapy, IL-6 pathway blockade significantly reduced intestinal damage whereas improved therapeutic outcomes in liver cancer patients (26). Anti-IL-6 receptor (anti-IL-6R) antibody, such as tocilizumab or sarilumab, achieved symptom resolution in approximately 73% irAEs cases among patients with melanoma, genitourinary cancer, or lung cancer in a retrospective analysis (27). Animal model studies of immune-related enterocolitis further showed that the IL-6 levels in intestinal tissues could initiate irAEs related colitis, while IL-6 inhibition simultaneously ameliorated neurotoxicity and enhanced antitumor immunity (28).

These consistent findings across clinical and experimental settings strongly suggest that IL-6 is involved in mediating both therapeutic response and irAEs development in cancer patients. However, the role of IL-6 on ICIs treatment for gastric adenocarcinoma (GAC) and esophageal squamous cell carcinoma (ESCC) — two of the most prevalent and aggressive upper gastrointestinal malignancies — remains unclear. To explore the relationship between IL-6 and irAEs occurrence in GAC and ESCC patients, we conducted the present analysis.

## 2 Methods

### 2.1 Patients

A total of 121 patients (87 male and 34 female) with a mean age of 64.1 ± 9.74 years were included in this study. The cohort comprised 79 patients with GAC and 42 with ESCC, who received anti-PD-1 therapy at the Fourth Hospital of Hebei Medical University between March 1, 2020, and August 31, 2023 were retrospectively reviewed. Inclusion criteria were as follows: (i) pathologically confirmed GAC or ESCC; (ii) unresectable patients with stage III or IV; (iii) completion of ≥2 cycles of PD-1 ICIs. Exclusion criteria included: (i) Missing clinical information; (ii) Prior receipt of other immunotherapies; (iii) Patients with successful conversion of neoadjuvant therapy to surgery; (iv) suffering from infection or rheumatic immune disease.

**Figure 1:**
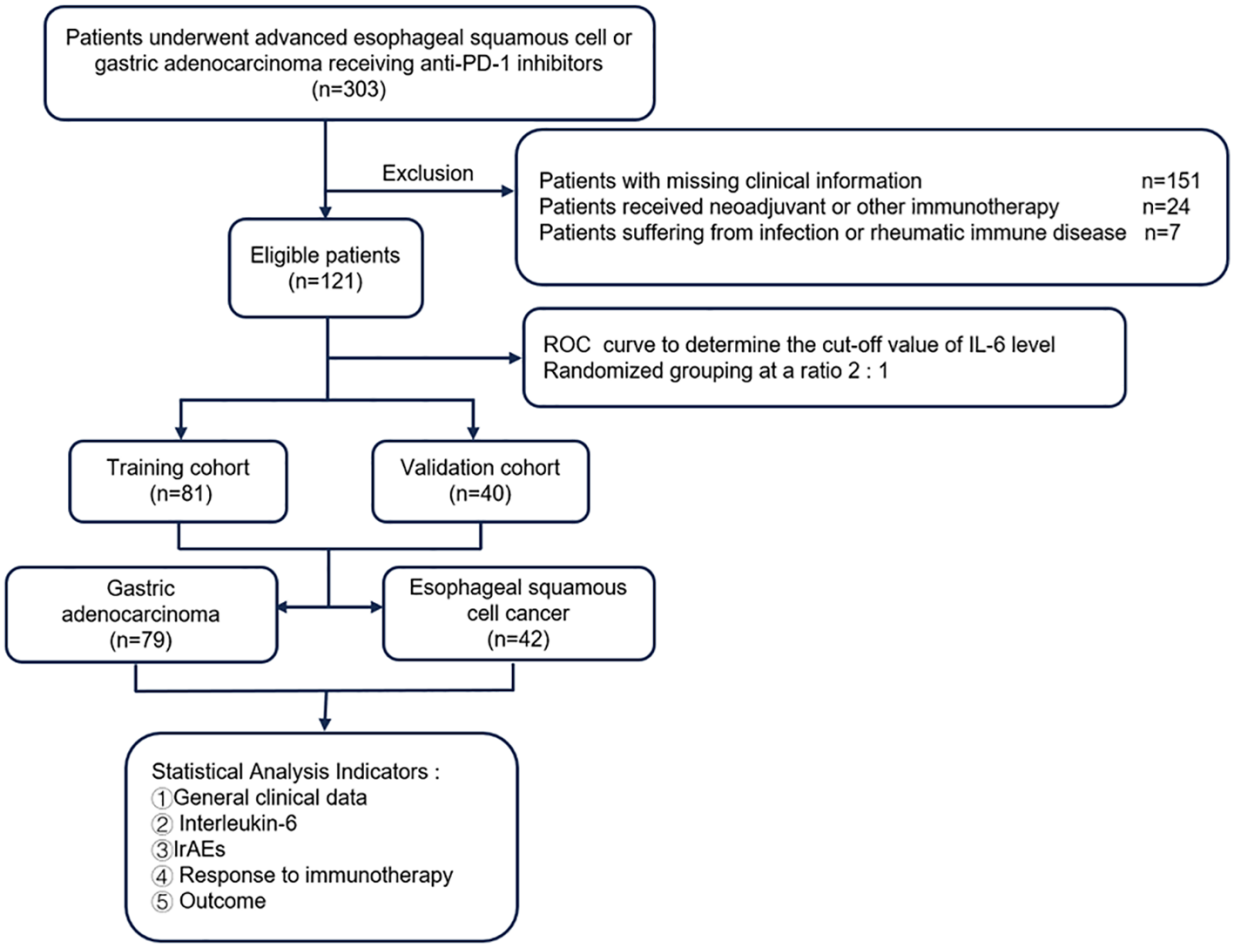
Flow diagram of the study. PD-1, programmed cell death 1; IL-6, interleukin-6; ROC, operating characteristic curve.

The entire cohort was initially divided into a training cohort (TC, n=81) and a validation cohort (VC, n=40) at a 2:1 ratio. Subsequently stratification was performed according to tumor type (GAC, n=79; ESCC, n=42) for individual tumor analysis. The following clinical parameters were systematically evaluated: gender, age, IL-6 levels included baseline or posttreatment, Eastern Cooperative Oncology Group performance status (ECOG PS), TNM stage (III/IV), surgery history (defined as cases of postoperative recurrence or metastasis), ICIs regimen, treatment lines, irAEs and cancer type. The flow chart of the analysis design is shown in **Figure 1**. IrAEs were defined as inflammatory toxicity caused by immune tolerance imbalance due to ICIs. The National Cancer Institute Common Terminology Criteria for Adverse Events ver.4.03 (http://ctep.cancer.gov/protocolDevelopment/elec-tronic_applications/ctc.htm#ctc_40) was used for the irAEs assessment. Given that grade 1 irAEs are generally asymptomatic, while grade ≥ 2 irAEs may cause symptoms and even lead to suspension or permanent discontinuation of ICIs, we stratified the irAEs cohort into grade 1 and grade ≥ 2 groups.

The timing of serum IL-6 measurements varied among participants, with some samples collected at baseline, others during treatment, and some at both baseline and post-treatment, including instances when irAEs occurred. For analysis purpose, we categorized the IL-6 level fluctuations as follows: if the overall change in IL-6 levels from baseline to post-treatment was no more than half of the initial value, we recorded the average of these levels. Conversely, if the fluctuation exceeded half of the initial level, we documented the highest value observed. Therefore, an increase in IL-6 either at the beginning or during the treatment process was defined as high IL-6. Boxplot analysis revealed 5 outliers in IL-6 levels among 121 patients, all of which were confirmed to be clinically relevant and thus retained. Specifically, 4 patients with GAC exhibited elevated IL-6 levels, concurrently presenting with irAEs. The types of irAEs observed included hepatotoxicity (n = 4), cardiotoxicity (n = 3), endocrine toxicity (n = 3), and dermatologic toxicity (n = 1). Additionally, 1 patient with ESCC demonstrated multi-organ endocrine toxicity involving the pituitary, thyroid, and adrenal glands. Comprehensive clinical profiles were available for all cases, confirming the biological significance of these outliers. These data points were preserved to ensure both clinical relevance and analytical rigor.

**Figure 2:**
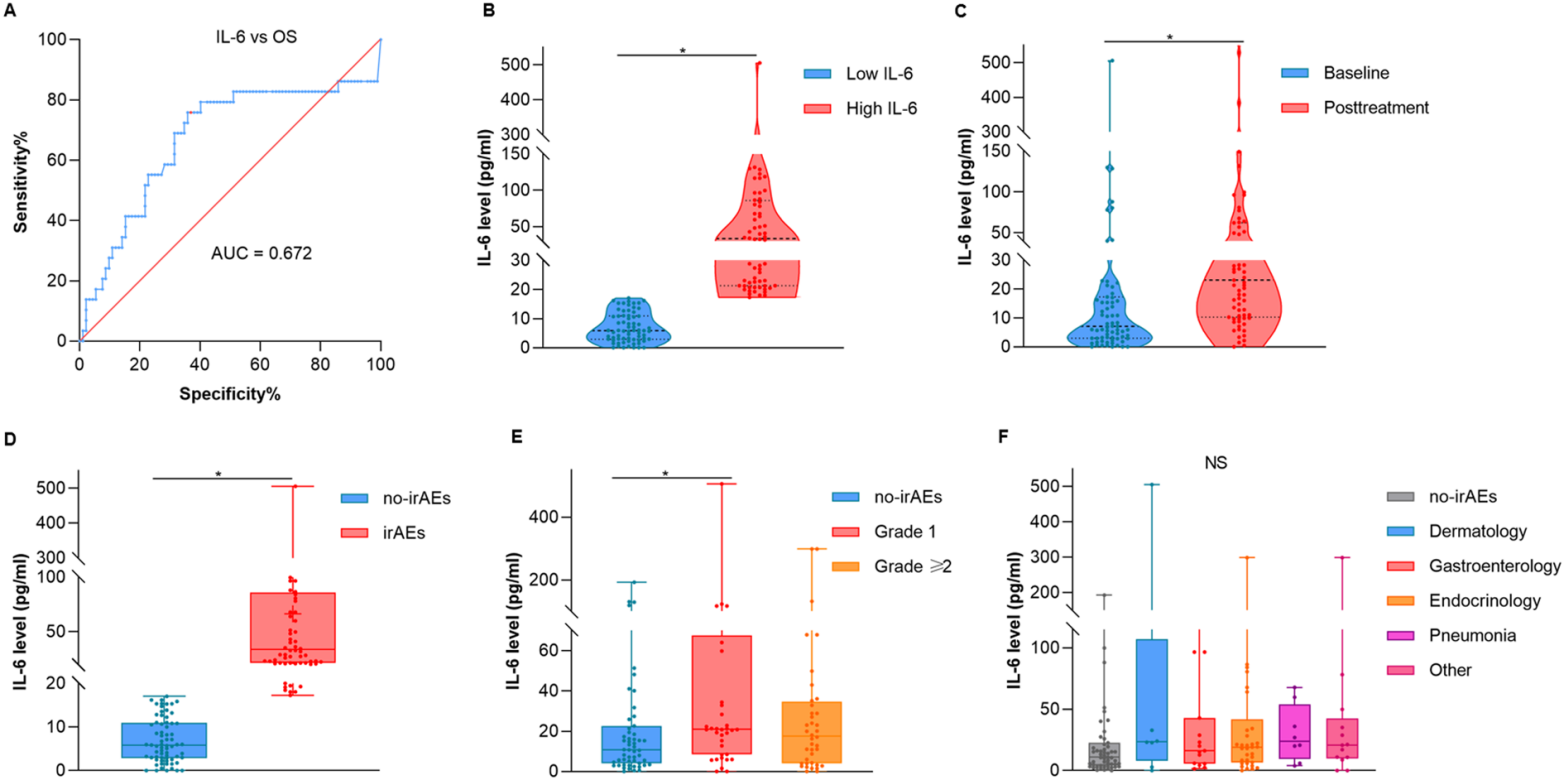
The correlation between the overall distribution of interleukin-6 and irAEs. **(A)** ROC curves for the serum IL-6 levels and OS. **(B)** Violin plot of the Low IL-6 and High IL-6 group. **(C)** Violin plot of the IL-6 at baseline and posttreatment. **(D)** Box and whisker diagram of IL-6 between no-irAEs and irAEs. **(E)** Box and whisker diagram of IL-6 across different grades. **(F)** Box and whisker diagram of IL-6 across different types of irAEs. OS, overall survival; AUC, area under curve. *p < 0.05.

To ascertain the optimal cut-off value for serum interleukin-6 (IL-6) levels, we utilized the receiver operating characteristic (ROC) curve analysis, which included OS as a parameter. Before ROC analysis, we performed Z-score normalization on the raw data using SPSS 25.0 software (IBM SPSS, NY, USA). Following transformation, the normalized data exhibited a mean of 0 and a standard deviation of 1, conforming to a standard normal distribution. We then generated the ROC curve (**Figure 2A**). The area under the curve (AUC) was calculated to be 0.672 (95% CI: 0.546–0.798, p=0.005). At the maximum Youden index (0.4), the optimal IL-6 cutoff was determined to be 17.16 pg/mL, which corresponded to a sensitivity of 75.9% and specificity of 64.1%. Thereby, the optimal cut-off value of IL-6 with 17.16 pg/ml was applied to distinguish patients as low IL-6 expression group (Low IL-6) and high IL-6 expression group (High IL-6).

Due to this retrospective study only utilized the existing anonymously information for analysis, a waiver of informed consent was applied for patients involved. All procedures performed in this study were in accordance with the Declaration of Helsinki and approved by the Ethics Committee of the Fourth Hospital of Hebei Medical University (No. 2024KS059).

### 2.2 Multiple microsphere flow immunofluorescence luminescence method

Serum IL-6 levels were determined using the Cytokine Detection Kit (Risker Biological Technology Co., Ltd. Qingdao, China), operated strictly according to the instructions by the laboratory department of our hospital. The cytokine antibody with fluorescent microsphere was combined with both the biotin-labeled cytokine pairing antibody and the cytokines in the sample to form a “sandwich” complex, which was subsequently reacted with the phycoerythrin-labeled streptavidin. The fluorescence intensity was detected by Navios flow cytometry (Beckman Coulter, Inc. Bria, California, USA). An eight points curve was drawn based the mean fluorescence intensity of the standard IL-6 values, which were diluted four times in sequence from 10000pg/ml (2500pg/ml, 625pg/ml, 156.3pg/ml, 39.1pg/ml, 9.8pg/ml, 2.4pg/ml, 0pg/ml). The IL-6 concentration of the samples was obtained by the position of their fluorescence intensity on the eight-points standard curve. IL-6 levels were measured before the treatment as well as after at least twice cycles of the treatment, with additional measurements taken depending on the treatment duration.

### 2.3 Treatment assessment

Patients received standard anti-PD-1 antibodies (mono-immunotherapy, in combination with chemotherapy or targeted drugs, or as triple therapy for combining ICIs with both chemotherapy and targeted therapy every 3 weeks until disease progression, clinical deterioration, intolerable toxicity or patient rejection. The types of immunotherapy drugs used included camrelizumab, sintilimab, pembrolizumab, toripalimab, serplulimab and tislelizumab, while the targeted were apatinib, regorafenib, trastuzumab and lenvatinib. Objective tumor response was assessed according to the “Response Evaluation Criteria in Solid Tumors” (RECIST) version 1.1 (29), using repeated computed tomography (CT) or magnetic resonance imaging (MRI) scans every 2 or 3 cycles.

### 2.4 Statistical analysis

Tumor effectiveness was evaluated based on ORR and (disease control rate) DCR according to RECIST version 1.1. PFS was defined as the time from first beginning of anti-PD-1 therapy to progression, death or study cutoff. OS was defined as the time from commencement of ICIs-based systemic therapy to death or study cutoff. GraphPad Prism version 8.0 (GraphPad Software, San Diego, California USA) was used to draw the graphs. All collected data were statistically analyzed by SPSS 25.0 software (IBM SPSS, NY, USA). Wilcoxon and Kruskal-Wallis tests were used for nonparametric rank sum test to explore differences between two dependent samples and among multiple groups. Clinical categorical count data were analyzed by chi-square test or Fisher’s exact test. Survival rates were estimated by Kaplan–Meier curve and Log-rank test. Univariate and multivariable COX regression analyses were performed to identify potential prognostic factors, with p < 0.05 considered statistically significant.

## 3 Results

### 3.1 The overall description of IL-6 and irAEs

A total of 121 patients were enrolled in the study. As shown in **Figure 2**, violin plots were employed to delineate the mean and density distribution disparities of IL-6 levels across all participants (**Figure 2B**, p = 0.022). A statistically significant elevation in IL-6 levels was observed post anti-PD-1 therapy when compared to baseline values (**Figure 2C**, p = 0.019).

Further analysis using the Wilcoxon test revealed that patients experiencing irAEs exhibited higher IL-6 levels than those without irAEs (**Figure 2D**, p = 0.005). Additionally, the Kruskal-Wallis test indicated that serum IL-6 levels were markedly elevated in patients with grade 1 irAEs compared to those without any irAEs (**Figure 2E**, p = 0.034). However, no significant correlation was observed between IL-6 levels and the specific types of irAEs encountered (**Figure 2F**, p = 0.321). **Table 1** presents a breakdown of the various irAEs, with each entry representing the count of patients affected by particular irAEs. It is noteworthy that the total count of irAEs types (n = 82) exceeds the total number of patients with irAEs (n = 65), as some individuals presented with multiple types of irAEs. These findings suggested that elevated serum IL-6 levels serve as a biomarker for the development of adverse events in patients receiving anti-PD-1 therapy, rather than being indicative of a specific type of irAEs.

**Table 1:** Types of irAEs and the number of patients by grade.

Grade	Type of irAEs	Counts of patients (n=65)
G1	Dermal toxicity	1
	Endocrine toxicity	
	Hypophysitis	12
	Thyroiditis	14
	Adrenocortical insufficiency	1
	Hepatotoxicity	5
	Gastrointestinal toxicity	1
	Pulmonary toxicity	3
	Muscle toxicity	1
	Cardiac toxicity	2
≥G2	Dermal toxicity	5
	Endocrine toxicity	
	Hypophysitis	7
	Thyroiditis	4
	Adrenocortical insufficiency	1
	Hepatotoxicity	5
	Gastrointestinal toxicity	4
	Pulmonary toxicity	5
	Muscle toxicity	2
	Cardiac toxicity	6
	Others (nephro, blood, nerves)	3

### 3.2 Internal validation of the correlation between IL-6 and the irAEs occurrence and patients’ prognosis

A total of 121 patients, including 79 with GAC and 42 with ESCC, were randomly divided into TC (n = 81) and VC (n = 40) groups at a 2:1 ratio. Baseline characteristics, including gender, age, ECOG score, tumor stage, surgery history, therapy regimen, and treatment lines, were well balanced between groups (**Table 2**).

**Table 2:** Baseline Characteristics of patients overall.

Variables	Training cohort				Validation cohort				p
	Total	Low IL-6	High IL-6	p	Total	Low IL-6	High IL-6	p	
Total	81	40	41		40	26	14		0.105
Gender									
Female	24 (29.6)	14 (35.0)	10 (24.4)		10 (25.0)	7 (26.9)	3 (21.4)		
Male	57 (70.4)	26 (65.0)	31 (75.6)	0.296	30 (75.0)	19 (73.1)	11 (78.6)	1.000	0.594
Age									
≥ 65	45 (55.6)	16 (40.0)	20 (48.8)		22 (55.0)	14 (53.8)	8 (57.1)		
< 65	36 (44.4)	24 (60.0)	21 (51.2)	0.427	18 (45.0)	12 (46.2)	6 (42.9)	0.842	0.954
ECOG PS									
≤ 2	73 (90.1)	35 (87.5)	38 (92.7)		31 (77.5)	18 (69.2)	13 (92.9)		
≥ 3	8 (9.9)	5 (12.5)	3 (7.3)	0.682	9 (22.5)	8 (30.8)	1 (7.1)	0.190	0.060
TNM									
III	35 (43.2)	21 (52.5)	14 (34.1)		18 (45.0)	12 (46.2)	6 (42.9)		
IV	46 (56.8)	19 (47.5)	27 (65.9)	0.095	22 (55.0)	14 (53.8)	8 (57.1)	0.842	0.852
Surgery history									
No	51 (63.0)	26 (65.0)	25 (61.0)		29 (72.5)	19 (73.1)	10 (71.4)		
Yes	30 (37.0)	14 (35.0)	16 (39.0)	0.708	11 (27.5)	7 (26.9)	4 (28.6)	1.000	0.297
Therapy									
ICIs monotherapy	1 (1.2)	0 (0.0)	1 (2.4)		1 (2.5)	1 (3.8)	0 (0.0)		
ICIs & chemotherapy	61 (75.3)	30 (75.0)	31 (75.6)		30 (75.0)	19 (73.1)	11 (78.6)		
ICIs & targeted	8 (9.9)	4 (10.0)	4 (9.8)		5 (12.5)	3 (11.5)	2 (14.3)		
Triple therapy	11 (13.6)	6 (15.0)	5 (12.2)	0.778	4 (10.0)	3 (11.5)	1 (7.1)	0.848	0.871
Cancer Type									
ESSC	30 (37.0)	17 (42.5)	13 (31.7)		12 (30.0)	7 (26.9)	5 (35.7)		
GAC	51 (63.0)	23 (57.5)	28 (68.3)	0.315	28 (70.0)	19 (73.1)	9 (64.3)	0.828	0.444
Treatment lines									
1 - 2	71 (87.7)	36 (90.0)	35 (85.4)		35 (87.5)	24 (92.3)	11 (78.6)		
≥ 3	10 (12.3)	4 (10.0)	6 (14.6)	0.767	5 (12.5)	2 (7.7)	3 (21.4)	0.452	0.981
irAEs									
No	39 (48.1)	25 (62.5)	14 (34.1)		17 (42.5)	14 (53.8)	3 (21.4)		
Yes	42 (51.9)	15 (37.5)	27 (65.9)	0.011*	23 (57.5)	12 (46.2)	11 (78.6)	0.048*	0.558

ECOG PS, Eastern Cooperative Oncology Group performance status; ICIs, Immune checkpoint inhibitors; Triple therapy, immunotherapy combination chemotherapy with targeted therapy; ESSC, esophageal squamous cell carcinoma; GAC, gastric adenocarcinoma; irAEs, immune-related adverse events. *p < 0.05.

**Figure 3:**
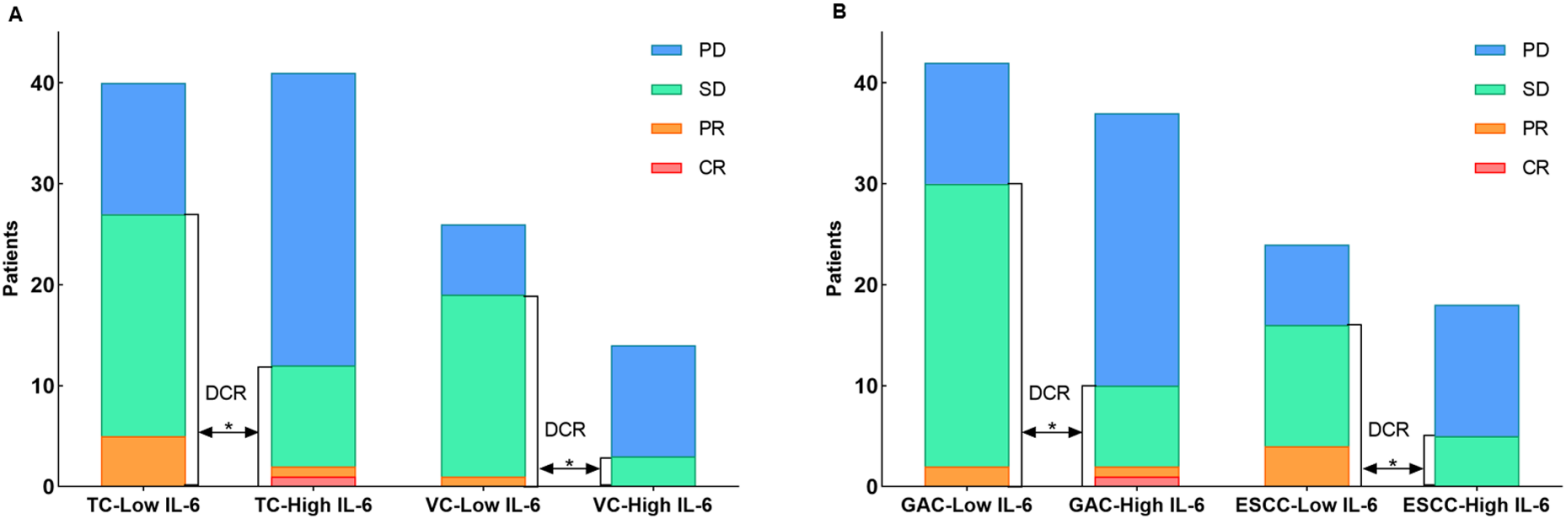
**(A)** The distribution of immunotherapy responses in the TC and VC groups. **(B)** The distribution of immunotherapy responses in the GAC and ESCC groups.

**Figure 4:**
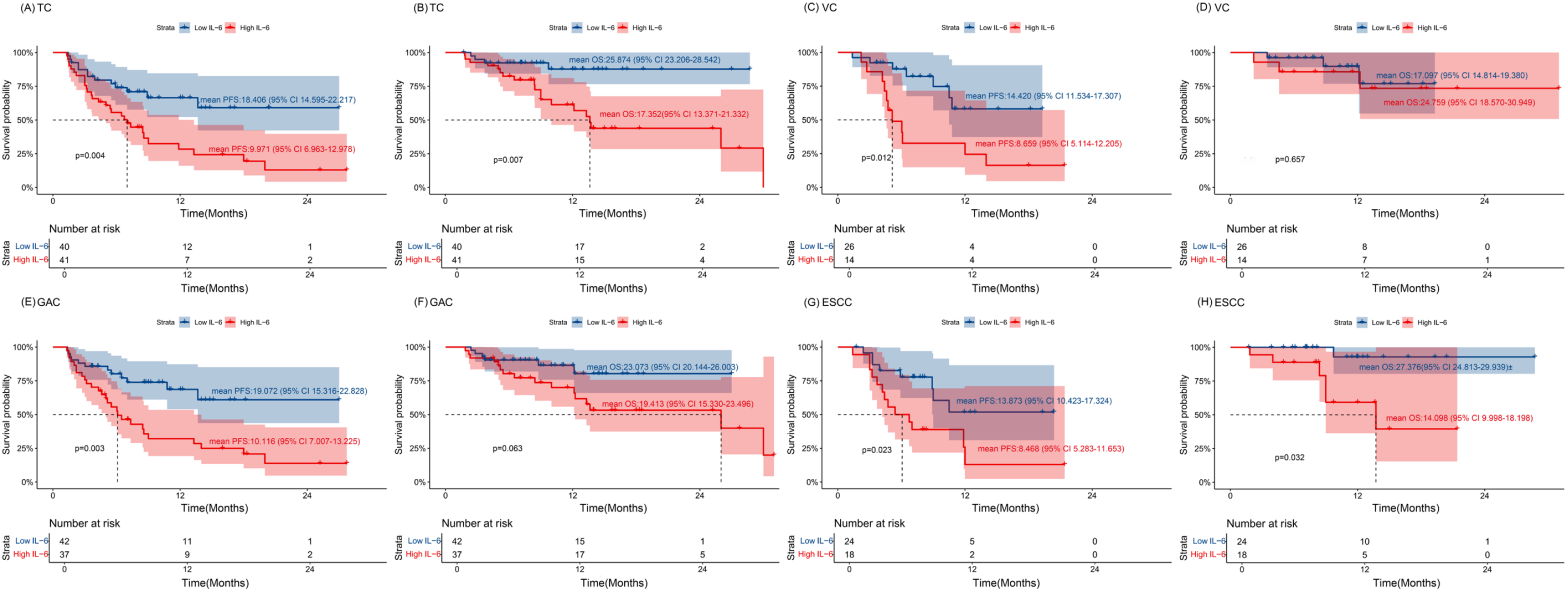
The association of IL-6 with the prognosis of overall patients **(A, B)** The Kaplan–Meier curve of PFS and OS for the TC cohort. **(C, D)** The Kaplan–Meier curve of PFS and OS for the VC cohort. **(E, F)** The Kaplan–Meier curve of PFS and OS for the GAC group. **(G, H)** The Kaplan–Meier curve of PFS and OS for the ESCC group. Time: Months; TC, training cohort; VC, validation cohort; ESCC, esophageal squamous cell carcinoma; GAC, gastric adenocarcinoma.

In TC group, higher IL-6 levels were associated with a higher incidence of irAEs (p = 0.011, **Table 2**). Outcome analysis also indicated high IL-6 were associated with lower DCR (29.3%, 95% CI: 14.7% - 43.8% vs. 67.5%, 95% CI: 52.3% - 82.7%, p = 0.001), shorter PFS (p = 0.004) and shorter OS (p = 0.007) when compared with those of low IL-6 group (**Figures 3A**, **4A, B**). Univariate COX regression analysis revealed the clinical characteristics, including higher IL-6, later TNM stage and later treatment lines were associated with worse outcome, including shorter PFS and shorter OS, in TC group (**Table 3**). Multivariable analysis indicated that high IL-6 levels and late treatment lines were independent risk factors modifying both PFS (HR = 2.102, 95% CI: 1.077 - 4.103, p = 0. 029; HR = 6.601, 95% CI: 3.042 - 14.321, p = 0. 000) and OS (HR = 3.309, 95% CI: 1.027 - 10.660, p = 0. 045; HR = 13.468, 95% CI: 3.752 - 48.342, p = 0. 000) (**Table 3**).

**Table 3:** Univariate and multivariable Cox proportional hazards model analyses of PFS and OS in TC group.

Variables	PFS				OS			
	Univariate		Multivariate		Univariate		Multivariate	
	HR (95%CI)	p	HR (95%CI)	p	HR (95%CI)	p	HR (95%CI)	p
High vs. Low IL-6	2.594 (1.345-5.002)	0.004*	2.102 (1.077-4.103)	0.029*	4.437 (1.500-13.123)	0.007*	3.309 (1.027-10.660)	0.045*
Male vs. Female	1.490 (0.741-2.994)	0.263			1.471 (0.570-3.795)	0.425		
Age ≥ 65 vs.< 65	0.983 (0.535-1.809)	0.957			1.008 (0.431-2.357)	0.986		
ECOG PS 3 vs. ≤ 2	0.429 (0.103-1.786)	0.245			0.509 (0.068-3.803)	0.510		
TNM IV vs. III	2.600 (1.343-5.035)	0.005*	1.813 (0.911-3.610)	0.090	3.665 (1.346-9.977)	0.011*	2.904 (0.812-10.384)	0.101
Surgery vs. no	0.564 (0.294-1.085)	0.086			0.690 (0.284-1.676)	0.412		
IrAEs vs. non-irAE	1.080 (0.585-1.992)	0.806			0.749 (0.324-1.732)	0.499		
ICIs monotherapy	Reference				Reference		Reference	
ICIs & chemotherapy	0.153 (0.020-1.179)	0.072			0.052 (0.006-0.452)	0.007*	0.094 (0.010-0.875)	0.038*
ICIs & targeted	0.468 (0.054-4.041)	0.490			0.341 (0.037-3.123)	0.341	0.048 (0.004-0.619)	0.020*
Triple therapy	0.303 (0.036-2.537)	0.270			0.086 (0.008-0.879)	0.039	0.078 (0.007-0.917)	0.042*
Treatment line ≥ 3	8.943 (4.155-19.251)	0.000*	6.601 (3.042-14.321)	0.000*	11.529 (4.715-28.190)	0.000*	13.468 (3.752-48.342)	0.000*
GAC vs. ESSC	0.912 (0.480-1.731)	0.778			0.983 (0.397-2.430)	0.970		

ECOG PS, Eastern Cooperative Oncology Group performance status; ICIs, Immune checkpoint inhibitors; Triple therapy, immunotherapy combination chemotherapy with targeted therapy; ESSC, esophageal squamous cell carcinoma; GAC, gastric adenocarcinoma; irAEs, immune-related adverse events; HR, hazard ratio; CI, confidence interval. *p < 0.05.

The association between high IL-6 levels and irAEs was confirmed in the VC group referring to the DCR (21.4%, 95% CI: 3.2% - 46.0% vs. 73.1%, 95% CI: 54.8% - 91.3%, p = 0.002) and PFS (p = 0.012) (**Figures 3A**, **4C, D**). Univariate COX regression (HR = 3.408, 95% CI: 1.303 - 8.911, p = 0. 012) and multivariable analysis (HR = 3.031, 95% CI: 1.110 - 8.276, p = 0. 030) showed that high IL-6 levels was significantly associated with shorter PFS, but no association was observed between IL-6 levels and OS (**Supplementary Table S1**). These data underscore that high IL-6 levels were not only associated with the occurrence of irAEs, but also the outcomes of ICIs treatment.

### 3.3 Individual analysis of gastric and esophageal carcinoma

Subsequent individual tumor analysis was performed for all GAC and ESCC patients in the VC and TC groups, and their characteristics are shown in **Table 4**. High IL-6 levels were confirmed to be linked with irAEs occurrence (p = 0.028), as well as a lower DCR (27.0%, 95% CI: 12.0% - 42.0% vs. 71.4%, 95% CI: 57.2% - 85.7%, p = 0.000), shorter PFS (p = 0.003) and a trend toward shorter OS (p = 0.063) in the GAC group (**Figures 3B**, **4E, F**). After univariate analysis, multivariable analysis identified high IL-6 levels (HR: 2.371, 95%CI: 1.086 - 5.179, p = 0.014) as an independent predictor for shorter PFS in GAC patients (**Table 5**). Consistent with the findings in GAC patients, higher IL-6 levels were also associated with irAEs occurrence (**Table 4**, p = 0.026) and lower DCR in ESCC patients (**Figure 3B**, p = 0.013). Survival analysis revealed associations with shorter PFS (p = 0.023) and OS (p = 0.032), and high IL-6 levels were verified as independent risk factors (**Figures 4G, H**, **Supplementary Table S2**). These data confirmed that the serum IL-6 levels were associated with both irAEs occurrence and treatment effectiveness in GAC and ESCC patients. We further evaluated the pathological characteristics, including HER2, MMR and PD-L1 status, for their association with IL-6 or irAEs in GAC and ESCC patients, but no statistically significant difference could be archived (data not shown).

**Table 4:** Characteristics of the patients with gastric adenocarcinoma and esophageal squamous cell cancer.

Variables	Gastric adenocarcinoma				Esophageal squamous cell carcinoma			
	Total	Low IL-6	High IL-6	p	Total	Low IL-6	High IL-6	p
Total	79	42	37		42	24	18	0.676
Gender								
Female	24 (30.4)	15 (35.7)	9 (24.3)		10 (23.8)	6 (25.0)	4 (22.2)	
Male	55 (69.6)	27 (64.3)	28 (75.7)	0.272	32 (76.2)	18 (75.0)	14 (77.8)	1.000
Age								
≥ 65	42 (53.2)	20 (45.0)	17 (45.9)		25 (59.5)	16 (66.7)	9 (50.0)	
< 65	37 (46.8)	22 (55.0)	20 (54.1)	0.882	17 (40.5)	8 (33.3)	9 (50.0)	0.276
ECOG PS								
≤ 2	64 (81.0)	31 (73.8)	33 (89.2)		40 (95.2)	22 (91.7)	18 (100)	
≥ 3	15 (19.0)	11 (26.2)	4 (10.8)	0.082	2 (4.8)	2 (8.3)	0 (0.0)	0.601
TNM								
III	29 (36.7)	21 (50.0)	8 (21.6)		24 (57.1)	12 (50.0)	12 (66.7)	
IV	50 (63.3)	21 (50.0)	29 (78.4)	0.009*	18 (42.9)	12 (50.0)	6 (33.3)	0.280
Surgery history								
No	51 (64.6)	26 (61.9)	25 (67.6)		29 (69.0)	19 (79.2)	10 (55.6)	
Yes	28 (35.4)	16 (38.1)	12 (32.4)	0.600	13 (31.0)	5 (20.8)	8 (44.4)	0.101
Thrapy								
ICIs monotherapy	0 (0.0)	0 (0.0)	0 (0.0)		2 (4.8)	1 (4.2)	1 (5.6)	
ICIs & chemotherapy	53 (67.1)	27 (64.3)	26 (70.3)		38 (90.5)	22 (91.7)	16 (88.9)	
ICIs & targeted	12 (15.2)	7 (16.7)	5 (13.5)		1 (2.4)	0 (0.0)	1 (5.6)	
Triple therapy	14 (17.7)	8 (19.0)	6 (16.2)	0.851	1 (2.4)	1 (4.2)	0 (0.0)	0.545
Treatment lines								
1-2	67 (84.8)	37 (88.1)	30 (81.1)		39 (92.9)	23 (95.8)	16 (88.9)	
≥3	12 (15.2)	5 (11.9)	7 (18.9)	0.386	3 (7.1)	1 (4.2)	2 (11.1)	0.795
irAEs								
No	36 (45.6)	24 (57.1)	12 (32.4)		20 (47.6)	15 (62.5)	5 (27.8)	
Yes	43 (54.4)	18 (42.9)	25 (67.6)	0.028*	22 (52.4)	9 (37.5)	13 (72.2)	0.026*

ECOG PS, Eastern Cooperative Oncology Group performance status; ICIs, Immune checkpoint inhibitors; Triple therapy, immunotherapy combination chemotherapy with targeted therapy; irAEs, immune-related adverse events; HR, hazard ratio; CI, confidence interval. *p < 0.05.

**Table 5:** Univariate and multivariable Cox proportional hazards model analyses of PFS and OS in gastric adenocarcinoma group.

Variables	PFS				OS			
	Univariate		Multivariate		Univariate		Multivariate	
	HR (95%CI)	p	HR (95%CI)	p	HR (95%CI)	p	HR (95%CI)	p
High vs. Low IL-6	2.879 (1.450-5.716)	0.003*	2.371 (1.086-5.179)	0.014*	2.474 (0.953-6.419)	0.063	1.541 (0.548-4.333)	0.412
Male vs. Female	0.769 (0.399-1.482)	0.432			1.045 (0.421-2.593)	0.924		
Age ≥ 65 vs. < 65	1.149 (0.606-2.180)	0.670			1.230 (0.508-2.976)	0.646		
ECOG PS 3 vs. ≤ 2	0.725 (0.280-1.878)	0.507			1.506 (0.495-4.583)	0.471		
TNM IV vs. III	2.285 (1.084-4.819)	0.030	1.010 (0.431-2.363)	0.988	3.514 (1.034-11.940)	0.044*	1.858 (0.470-7.351)	0.377
Surgery vs. no	0.333 (0.156-0.714)	0.005	0.175 (0.072-0.428)	0.000*	0.335 (0.111-1.009)	0.052		
IrAEs vs. non-irAE	0.901 (0.473-1.717)	0.752			0.405 (0.167-0.982)	0.045*	0.598 (0.202-1.769)	0.353
ICIs & chemotherapy	Reference		Reference		Reference		Reference	
ICIs & targeted	3.381 (1.522-7.509)	0.003*	1.366 (0.365-5.117)	0.552	5.517 (2.207-13.787)	0.000*	0.943 (0.132-6.735)	0.953
Triple therapy	2.214 (1.031-4.756)	0.042*	2.230 (0.869-5.723)	0.092	0.855 (0.230-3.178)	0.815	0.374 (0.071-1.981)	0.247
Treatment line ≥ 3	5.159 (2.522-10.553)	0.000*	11.039 (4.150-29.367)	0.011*	7.024 (2.955-16.696)	0.000*	5.686 (0.887-36.428)	0.067

PFS, progression free survival; OS, overall survival; ECOG PS, Eastern Cooperative Oncology Group performance status; irAEs, immune-related adverse events; ICIs, Immune checkpoint inhibitors; Triple therapy, immunotherapy combination chemotherapy with targeted therapy; HR, hazard ratio; CI, confidence interval. *p < 0.05.

## 4 Discussion

Our data demonstrated that higher levels of serum IL-6 were associated with both irAEs occurrence and treatment effectiveness (DCR, PFS and OS) in patients receiving ICIs in TC cohort. These potential associations also confirmed in VC cohort, except for OS. Several factors, including insufficient sample size limited statistical power to detect meaningful differences, potential influence of pathological characteristics, comorbidities and follow-up duration, might have modified the final survival analysis. The consistent statistical significance of PFS across all cohorts strongly suggests that early elevation of IL-6 may serve as a timely biomarker of immunotherapy response. Individual tumor analysis in both GAC and ESCC patients also confirmed the results observed in TC and VC analyses. Our study is consistent with previous reports that higher IL-6 levels are correlated with the worse prognosis in non-small cell lung cancer (NSCLC) and melanoma patients (30–34).

The key signaling pathways of the IL-6/JAK/STAT3 axis promote tumor growth, metastasis, and metabolism (35), IL-6 also activates the Yes-associated protein (YAP) and nuclear factor kappa-light-chain-enhancer of activated B cells (NF-kB) signaling pathways to promote cell proliferation, migration and invasion as well as mediating activation of transcription factors CCAAT/enhancer-binding protein beta/delta (C/EBPβ/δ) to induce epithelial-mesenchymal transition (EMT) and amplification of cancer stem cells (36, 37). Beyond its pro-tumorigenic roles, IL-6 promotes angiogenesis by the VEGF signal pathway, thereby weakening the effectiveness of ICIs in many types of cancers, including gastrointestinal cancers, prostate cancer, oral squamous cell carcinoma, and hepatocellular carcinoma (38–40). A novel role of tumor-intrinsic PD-L1/JAK/STAT3/IL-6/MDSC axis in both immunosuppression and tumor progression has been recently reported in NSCLC (41). These mechanisms may partly explain the negative impact of IL-6 on ICIs treatment efficacy.

In the tumor microenvironment, cytokines generation is one of the main mechanisms underlying irAEs development (9). Preclinical studies with irAEs model have found that irAEs significantly induce IL-6 production (42). Clinically, elevated IL-6 levels have been associated with the occurrence of psoriatic dermatitis in patients with malignant melanoma receiving nivolumab therapy (43). These findings are consistent with our observations in GAC and ESCC patients, that IL-6 was positively associated with irAEs occurrence. The precise mechanism needs to be fully elucidated to determine which factor initially triggers the others. However, the therapeutic potential of IL-6 inhibition for irAEs has been explored. An anti-IL-6R monoclonal antibody tocilizumab has been applied in clinical treatment for irAEs, including colitis, arthritis and irAEs related cytokine release syndrome (44–47). All of above indicate that IL-6 is not only a target for tumor control but also a contributor to irAEs.

Our study showed that high serum IL-6 levels were associated with both irAEs occurrence and poor outcomes. However, previous reports and our own findings have indicated that irAEs are correlated with better treatment effectiveness in gastrointestinal tumors (48–50). IL-6 appears to exhibit dual effect on ICIs treatment, with implications for both treatment efficiency and irAEs. The mechanisms underlying irAEs-mediated ICIs effectiveness might be highly complex, involving multiple organs, including the lungs, gastrointestinal tract, thyroid, skin, joint, and so on. The different types and grades of irAEs toxicity might exert different effects on different tumors due to variations in the immune microenvironment (51). Previous reports have suggested that only gastrointestinal tract, thyroid and skin related irAEs are associated with better ICIs treatment efficiency (14, 49, 52, 53). We also found that cardiac, hepatic, and pulmonary irAEs displayed negative or neutral effects on ICIs efficiency. The small sample size limits our ability to further evaluation of which irAEs (positive, negative or neutral effect on ICIs efficiency) are related to IL-6 promotion. Additionally, many clinical characteristics, including age, ECOG PS score, TNM stage, and treatment line appear to contribute to ICIs effectiveness beyond irAEs in gastrointestinal tumors. The different tumor microenvironments of each individual, which are not clearly defined, can also affect the final outcome. All of these factors might contribute to the dual effect of IL-6. Further stratified analyses with larger sample size are needed to evaluate the effects of IL-6 on different types of irAEs and the prognostic correlation of different types of irAEs in cancer treatment (53, 54).

This study had some limitations. Firstly, it is retrospective study conducted in a single medical center with relatively small sample size, making it difficult to collect complete paired serum IL-6 data with standard spatial and temporal differences for statistical analysis. Secondly, this study did not completely rule out the effects of adverse reactions resulted from the combined target therapy and chemotherapy.

To our knowledge, this is one of the few real-world studies to reveal the relationship between IL-6 and the effectiveness of ICIs, focusing on gastric and esophageal cancer. These findings may guide us to identify irAEs as early as possible and minimize their adverse effects of irAEs on tumor treatment. Furthermore, the potential predictive value of IL-6 for irAEs and the effectiveness of ICIs treatment may pave the way for future prospective studies involving larger cohorts. These insights may further motivate other researchers to explore the predictive potential of IL-6 in ICIs treatment across a broader range of tumors. This could facilitate the development of more precise patient screening protocols and ultimately contribute to the optimization of the therapeutic benefits of ICIs.

In conclusion, our findings demonstrate that elevated IL-6 not only correlates with the incidence of irAEs but also serves as a prognostic indicator for poorer outcomes in gastric adenocarcinoma and esophageal squamous cell carcinoma patients receiving ICIs. These associations may extend to other malignancies of similar origin, such as colorectal cancer and hepatobiliary cancers. Further investigations are needed to validate IL-6 as both a predictive marker of irAEs occurrence and a treatment target for irAEs management.

## Data Availability

The original contributions presented in the study are publicly available. This data can be found here: https://doi.org/10.5281/zenodo.15332218.
