# Supplementary material for: Serum IL-6 predicts immunotherapy-related adverse and outcome in advanced gastric and esophageal cancer patients with Anti-PD-1 treatment
Source: Front Immunol. 2025 May 30;16:1553882. doi: 10.3389/fimmu.2025.1553882 (PMC12163003; doi:10.3389/fimmu.2025.1553882)
Supplement: Supplementary file 1 [file DataSheet1.docx]

upplementary Material

# Supplementary Tables and Figure

## Supplementary Tables

## Table S1 Univariate and multivariable Cox proportional hazards model analyses of PFS and OS in validation cohort.

| Variables | PFS | | | |  | OS | | | | |  |
| --- | --- | --- | --- | --- | --- | --- | --- | --- | --- | --- | --- |
|  | Univariate | | Multivariate | |  | Univariate | | Multivariate | | |  |
|  | HR (95%CI) | p | HR (95%CI) | p | | HR (95%CI) | p | | HR (95%CI) | p | |
| High vs. Low IL-6 | 3.408(1.303-8.911) | 0.012* | 3.031 (1.110-8.276) | 0.030* | | 1.444(0.286-7.304) | 0.657 | |  |  | |
| Male vs. Female | 0.550(0.206-1.468) | 0.232 |  |  | | 0.297(0.059-1.489) | 0.140 | |  |  | |
| Age ≥ 65 vs.< 65 | 1.672(0.623-4.489) | 0.307 |  |  | | 0.442(0.081-2.426) | 0.348 | |  |  | |
| ECOG PS 3 vs. ≤ 2 | 1.044(0.297-3.665) | 0.946 |  |  | | 4.320(0.867-21.536) | 0.074 | | 9.052(1.332-61.175) | 0.024* | |
| TNM Ⅳ vs. Ⅲ | 878(0.343-2.249) | 0.786 |  |  | | 48.938(0.054-44432.861) | 0.263 | |  |  | |
| Surgery vs. no | 0.416(0.120-1.448) | 0.168 |  |  | | 0.029(0.000-47.982) | 0.348 | |  |  | |
| IrAEs vs. non-irAE | 1.016(0.378-2.735) | 0.974 |  |  | | 0.449(0.086-2.331) | 0.341 | |  |  | |
| ICIs monotherapy | Reference |  |  |  | | Reference |  | |  |  | |
| ICIs & chemotherapy | 9198.724(0.0-2.293E+104) | 0.938 |  |  | | 2012.705(0.000-4.154E+110) | 0.952 | |  |  | |
| ICIs & targeted | 12838.503(0.0-1.070E+105) | 0.928 |  |  | | 27266.499(0.000-5.617E+110) | 0.935 | |  |  | |
| Triple therapy | 18331.999(0.0-4.578E+104) | 0.934 |  |  | | 1.020(0.000-1.604E+127) | 1.000 | |  |  | |
| Treatment line ≥ 3 | 2.733(0.894-8.360) | 0.078 | 1.772(0.551-5.697) | 0.337 | | 7.147(1.429-35.756) | 0.017* | | 0.072(0.011-0.477) | 0.006* | |
| GC vs. ESSC | 1.107(0.412-2.976) | 0.840 |  |  | | 39.912(0.035-45675.484) | 0.305 | |  |  | |

Abbreviations: PFS: progression free survival, OS: overall survival, ECOG PS: Eastern Cooperative Oncology Group performance status, irAEs, immune-related adverse events, ICIs: Immune checkpoint inhibitors, Triple therapy: immunotherapy combination chemotherapy with targeted therapy, GC: gastric cancer, ESSC: esophageal squamous cell carcinoma, HR: hazard ratio, CI: confidence interval. *p < 0.05.

## Table S2 Univariate and multivariable Cox proportional hazards model analyses of PFS and OS in esophageal squamous cell carcinoma group.

| Variables | PFS | | | | |  | OS | | | | | |
| --- | --- | --- | --- | --- | --- | --- | --- | --- | --- | --- | --- | --- |
|  | Univariate | | Multivariate | |  | | | Univariate | Multivariate | | | |
|  | HR (95%CI) | p | HR (95%CI) | p | | | HR (95%CI) | | | p | HR (95%CI) | p |
| High vs. Low IL-6 | 2.800(1.153-6.798) | 0.023* | 2.946 (1.203-56.002) | 0.018* | | | 10.260(1.224-85.990) | | | 0.032* | 74.440 (3.474-1595.073) | 0.006* |
| Male vs. Female | 2.779 (0.806-9.578) | 0.105 |  |  | | | 1.077(0.207-5.593) | | | 0.930 |  |  |
| Age ≥ 65 vs. < 65 | 1.239(0.499-3.076) | 0.644 |  |  | | | 0.306(0.059-1.596) | | | 0.160 |  |  |
| ECOG PS 3 vs. ≤ 2 | 0.044(0.000-128.201) | 0.443 |  |  | | | 0.043(0.000-5135.73) | | | 0.598 |  |  |
| TNM Ⅳ vs. Ⅲ | 1.755(0.720-44.279) | 0.216 |  |  | | | 7.770(1.447-41.719) | | | 0.017* | 55.671(3.440-900.983) | 0.005* |
| Surgery vs. no | 1.241(0.499-3.088) | 0.642 |  |  | | | 1.636(0.365-7.337) | | | 0.520 |  |  |
| IrAEs vs. non-irAE | 1.421(0.588-3.438) | 0.435 |  |  | | | 2.202(0.421-11.526) | | | 0.350 |  |  |
| ICIs monotherapy | Reference |  |  |  | | | Reference | | |  |  |  |
| ICIs & chemotherapy | 1.000(0.147-6.784) | 1.000 |  |  | | | 1.000(0.023-42.672) | | | 1.000 |  |  |
| ICIs & targeted | 1.000(0.000-435595) | 1.000 |  |  | | | 1.000(0.000-5381116.160) | | | 1.000 |  |  |
| Triple therapy | 1.000(0.012-84.443) | 1.000 |  |  | | | 1.000(0.004-231.459) | | | 1.000 |  |  |
| Treatment line ≥ 3 | 11.341(2.783-46.223) | 0.001* | 12.959(2.999-56.002) | 0.001* | | | 5.592(1.013-30.856) | | | 0.048* | 9.961(0.912-108.808) | 0.060 |

Abbreviations: PFS: progression free survival, OS: overall survival, ECOG PS: Eastern Cooperative Oncology Group performance status, irAEs: immune-related adverse events, ICIs: Immune checkpoint inhibitors, Triple therapy: immunotherapy combination chemotherapy with targeted therapy, HR: hazard ratio, CI: confidence interval. *p < 0.05.
